# Immunogenicity and safety of inactivated SARS-CoV-2 vaccine in haemodialysis patients: a prospective cohort study

**DOI:** 10.1038/s41598-023-38628-2

**Published:** 2023-07-18

**Authors:** Metalia Puspitasari, Prenali D. Sattwika, Dzerlina S. Rahari, Wynne Wijaya, Auliana R. P. Hidayat, Nyoman Kertia, Bambang Purwanto, Jarir At Thobari

**Affiliations:** 1 Department of Internal Medicine, Faculty of Medicine, Public Health and Nursing, Universitas Gadjah Mada/Dr. Sardjito General Hospital, Yogyakarta, Indonesia; 2 Clinical Epidemiology and Biostatistics Unit, Faculty of Medicine, Public Health and Nursing, Universitas Gadjah Mada/Dr. Sardjito General Hospital, Yogyakarta, Indonesia; 3 Cardiovascular Clinical Research Facility, Division of Cardiovascular Medicine, Radcliffe Department of Medicine, University of Oxford, Oxford, UK; 4 Department of Internal Medicine, Faculty of Medicine, Universitas Sebelas Maret, Surakarta, Indonesia; 5 Department of Pharmacology and Therapy, Faculty of Medicine, Public Health and Nursing, Universitas Gadjah Mada, Yogyakarta, Indonesia

**Keywords:** Adaptive immunity, Vaccines, Immunology, Nephrology, Kidney diseases, Chronic kidney disease

## Abstract

End-stage renal disease patients on haemodialysis (HD) have been largely excluded from SARS-CoV-2 vaccine trials due to safety reasons and shown to mount lower responses to vaccination. This study aims to evaluate the immunogenicity and safety of inactivated COVID-19 vaccine among HD patients compared to healthy controls. All subjects who received the primary inactivated COVID-19 vaccination had their blood samples tested 21 days after the second dose. We report the immunogenicity based on anti-RBD IgG titre (IU/mL), the inhibition rate of neutralizing antibodies (NAbs) (%) to RBD, and seroconversion rates. Adverse events were assessed within 30 min and on the 7th day after each dose. Among 75 HD patients and 71 healthy controls, we observed no significant difference in all immunogenicity measures: anti-RBD IgG GMT (277.91 ± 7.13 IU/mL vs. 315.50 ± 3.50 IU/mL, p = 0.645), NAbs inhibition rate (82% [53–96] vs. 84% [39–98], p = 0.654), and seroconversion rates (anti-RBD IgG: 86.7% vs. 85.9%, p = 0.895; NAbs: 45.3% vs. 60.6%, p = 0.065). The number of adverse events is not significantly different between the two groups. The primary inactivated SARS-CoV-2 vaccination elicits an adequate antibody response and can be safely administered in haemodialysis patients.

## Introduction

The coronavirus disease (COVID-19) pandemic has caused significant morbidity and mortality worldwide. The clinical presentation varies from asymptomatic and mild disease to pneumonia and respiratory failure^1^. Patients with end-stage renal disease (ESRD) requiring dialysis are identified as high-risk patients for a severe form of infection from severe acute respiratory syndrome coronavirus 2 (SARS-CoV-2) due to their frequent contacts with health care providers and other patients as well as their high burden of comorbid conditions^2^. Maintenance haemodialysis (HD) patients with Coronavirus Disease (COVID)-19 possess a higher incidence (7.7%) and mortality rate (20–25%) than the general population^3–6^. The high rate of incidence, morbidity, and mortality associated with ESRD calls for urgent measures to prevent life-threatening complications. COVID-19 vaccines were developed rapidly to control COVID-19 transmission and have shown remarkable efficacy in clinical trials and nationwide studies^7^.

End-stage renal disease patients, including HD patients, have been largely excluded from vaccine trials for safety reasons. Furthermore, ESRD patients mount lower responses to vaccination than healthy individuals due to dysfunction of the adaptive immune system^8–10^. Therefore, an effective COVID-19 vaccination strategy is crucial in this population. Inactivated COVID-19 vaccines were the most widely available vaccine platform in Indonesia in 2021 and were incorporated into the national mass vaccination program by the government, including for the ESRD population. Studies regarding the immunogenicity and safety of any COVID-19 vaccines in Indonesian haemodialysis patients have never been conducted. In this study, we aim to evaluate the immunogenicity and safety of inactivated COVID-19 vaccination among haemodialysis patients in comparison with healthy subjects in Yogyakarta and Central Java, Indonesia. We assess the immunogenicity by measuring anti-receptor binding domain (RBD) IgG, neutralizing antibodies (NAbs) median inhibition, and seroconversion rates.

## Methods

### Study design

**Figure 1:**
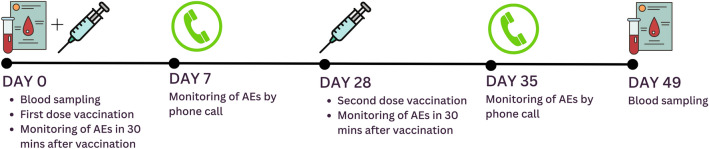
Study timeline.

An observational prospective cohort study was conducted among maintenance haemodialysis patients and healthy individuals who received the primary inactivated COVID-19 vaccination (CoronaVac®) during the national mass vaccination program in the Special Region of Yogyakarta and Central Java between July 2021 to October 2021. The timeline of this study is presented in Fig. 1. We recruited haemodialysis patients from four outpatient dialysis centres in Yogyakarta and Central Java, Indonesia. Subjects were vaccinated with two doses of inactivated SARS-CoV-2 vaccine (CoronaVac, 3 µg of inactivated whole-virus SARS-CoV-2 in 0.5 ml) 28 days apart. We evaluated the anti-RBD IgG antibody titre, inhibition rate (%inhibition) of NAbs to RBD of SARS-CoV-2, and seroconversion rates in HD patients compared to healthy controls. We also monitored and compared local and systemic adverse events in both groups. Seroconversion is defined as the conversion from seronegative (NAbs %inhibition < 30%) at baseline to seropositive (NAbs %inhibition ≥ 30%). Blood samples were collected twice: before vaccination and 21 days after the second dose. All participants were monitored for solicited and unsolicited AEs within 30 and on the 7th day after each dose by phone call.

### Patient selection

Generally, we included subjects aged between 18 and 59 years old and excluded those with symptoms of respiratory tract infections within the past week, evidence of ongoing infections, and history of COVID-19. HD patients were included if they had undergone routine haemodialysis twice weekly for at least three months with an arteriovenous (AV) fistula or graft as the vascular access. All HD patients in our dialysis centres undergo haemodialysis only twice weekly due to limited resources. Patients with double-lumen catheters (DLC) as the vascular access were excluded to acquire a homogenous sample. Moreover, AV fistula has been shown to contribute to better survival among HD patients with COVID-19^11^. Healthy subjects with a history of kidney diseases was excluded. Any subjects were excluded if they have acute conditions related to ESRD or other chronic diseases, symptoms or signs of respiratory tract infection or systemic infection during the study period, are taking steroid or immunosuppressive therapy (equivalent with methylprednisolone > 8 mg daily), or have previously received any COVID-19 vaccines.

### Anti-RBD IgG assay

Antibodies (IgG) against RBD of the spike protein of SARS-CoV-2 were measured with electrochemiluminescence immunoassays (ECLIA) (Elecsys Anti-SARS-CoV-2 S, Roche Diagnostics, Germany). Blood samples were incubated with a mix of biotinylated and ruthenylated RBD antigens. Double-antigen sandwich immune complexes (DAGS) are formed in the presence of corresponding antibodies. After adding streptavidin-coated microparticles, the DAGS complexes bind to the solid phase via the interaction of biotin and streptavidin. The reagent mixture is transferred to the measuring cell, where the microparticles are magnetically captured onto the surface of the electrode. Unbound substances are subsequently removed. Electrochemiluminescence is then induced by applying a voltage and measured with a photomultiplier. The signal yield increases with the antibody titre. According to the manufacturer’s guidelines, a titre of ≥ 0.8 U/mL is considered seropositive (sensitivity 98.8% and specificity 99.9%)^12^.

### Neutralizing antibodies (NAbs) tests

According to the manufacturer’s instructions, blood specimens were tested for NAbs against RBD using a surrogate viral neutralization test (sVNT) (cPass™, GenScript USA). Horseradish peroxidase (HRP)-RBD is pre-incubated with test serum (1:10 diluted) for 1 h at 37 °C. After that, it is added onto the ELISA plate pre-coated with hACE2 (GenScript). The unbound HRP-RBD is washed off, and bound RBD-ACE2 is detected colourimetrically. Circulating NAbs against SARS-CoV-2 competitively inhibits the RBD-ACE2 interaction. The percentage of inhibition is calculated by measuring the difference in the amount of labelled RBD between test versus control samples. The cut-off value for neutralizing antibodies is 30% signal inhibition^13^.

### Adverse events

Adverse events were categorized as local and systemic and classified into Grades 1 to 4 according to subjective severity (Table 1). Grade 1 AE represented mild symptoms (does not interfere with activity), Grade 2 entailed moderate symptoms (interferes with activity), Grade 3 entailed severe symptoms (prevents daily activity), and Grade 4 involved an emergency department visit or hospitalization. The grades were established according to the Food and Drug Administration toxicity grading scale^14^. The occurrence of adverse events was documented in an adverse events card.

**Table 1:** Baseline characteristics of subjects.

Characteristics	HD (n = 75)	Control (n = 71)	p value*
Gender, n (%)			
Male	38 (50.7%)	33 (46.5%)	0.613
Female	37 (49.3%)	38 (53.5%)	
Age, years			
Median (IQR)	45 (39–52)	43.21 (38–51)	0.658
Min–max	19–59	22–59	
Occupation, n (%)			
Office workers	31 (41.3%)	26 (36.6%)	0.560
Field workers	36 (48.0%)	39 (54.9%)	0.402
Students	1 (1.3%)	4 (5.6%)	0.200
No occupation/Retired	7 (9.3%)	2 (2.8%)	0.167
Comorbid conditions, n (%)			
Diabetes mellitus	12 (16%)	4 (5.6%)	**0.045**
Hypertension	65 (86.7%)	6 (8.3%)	** < 0.0001**
Coronary artery disease (CAD)	3 (4%)	0 (0%)	0.245
Stroke	3 (4%)	0 (0%)	0.245
Cancer	1 (1.3%)	0 (0%)	1.000
Hepatitis B	2 (2.7%)	1 (1.4%)	1.000
Hepatitis C	5 (6.7%)	0 (0%)	0.059
Tuberculosis	2 (2.7%)	1 (1.4%)	1.000
Anemia	37 (49.3%)	3 (4.2%)	** < 0.0001**
Autoimmune diseases	0 (0%)	0 (0%)	NA
Arthritis	17 (22.7%)	8 (11.3%)	0.068
Asthma	5 (6.7%)	5 (7.0%)	1.000
Allergic diseases	7 (9.3%)	4 (5.6%)	0.397
Laboratory values			
Hemoglobin, g/dL			
Mean ± SD	9.54 (1.57)	13.79 (1.88)	** < 0.0001**
Lymphocyte count, 10^9^ cells/L			
Mean ± SD	1.557.73 ± 649.81	2.213.52 ± 579.71	** < 0.0001**
Platelet count, 10^9^ cells/L			
Mean ± SD	206.080.00 ± 70.424.92	267.704.23 ± 56.031.47	** < 0.0001**
BUN, mg/dL			
Median (IQR)	58.20 (42.7–77.3)	10.20 (8.1–12.5)	** < 0.0001**
Creatinine, mg/dL			
Median (IQR)	10.98 (8.24–13.44)	0.85 (0.73–1.00)	** < 0.0001**
AST, U/L			
Median (IQR)	15 (10–20)	19 (15–23)	**0.001**
ALT, U/L			
Median (IQR)	14.5 (8–23)	18 (13–27)	**0.036**
Ferritin, ng/mL			
Median (IQR)	336.71 (105.74–798.41)	84.94 (50.30–206.71)	** < 0.0001**
Albumin, g/dL			
Mean ± SD	4.05 ± 0.51	4.51 ± 0.25	** < 0.0001**
Uric Acid, mg/dL			
Median (IQR)	6.6 (5.5–8.0)	4.9 (4.2–5.7)	** < 0.0001**
NAb, % inhibition			
≤ 30 (Seronegative)	42 (56%)	43 (60.6%)	0.576
> 30 (Seropositive)	33 (44%)	28 (39.4%)	

*Pearson’s chi-square or Fisher’s exact for categorical variables, independent t test or Mann Whitney U test for continuous variables, statistically significant if p value < 0.05 (typed in bold).

### Sample size calculation

The minimum sample size was determined using the software: Power and Sample Size Calculation program version 3.1.2 by William D. Dupont and Walton D. Plummer Jr. Means and standard deviations of anti-RBD IgG titres and NAbs from previous studies are needed to calculate the minimum sample size. For the independent group, n1 equals n2, α is 0.05, and the desired research power (1 – β) is 0.8. For anti-RBD IgG, Geisen et al. demonstrated an anti-RBD IgG level of 2053 ± 1218 BAU/mL in HD patients and 2685 ± 1102 BAU/mL in healthy controls^15^. Accordingly, the minimum sample size is 59 subjects for each group. For NAbs, Geisen et al. showed an inhibition rate of 87.42 ± 17.94% among HD patients and 96.04 ± 15.51% among healthy controls^15^. These yielded a minimum sample size of 69 subjects for each group.

### Statistical analysis

Demographic, clinical, and laboratory data were collected from all patients. Categorical and continuous variables were reported as absolute numbers, frequencies, means with standard deviation (SD), or medians with interquartile range (IQR). The normality of data distribution was tested with the Shapiro–Wilk test. Continuous variables were compared between HD patients and healthy controls using the independent t-test or Mann–Whitney test following its normality. Categorical variables were compared using Pearson’s chi-square or Fisher’s exact test. Statistical analyses were performed with IBM SPSS statistical software, version 26 (IBM, USA). Results were considered significant if the p-value < 0.05.

### Ethical considerations

The Medical and Health Research Ethics Committee of the Faculty of Medicine, Public Health, and Nursing, Universitas Gadjah Mada, Yogyakarta, Indonesia, approved the study protocol (No. KE-FK-0890-EC-2021). The study was conducted by the Declaration of Helsinki and Good Clinical Practice. All participants have been informed and have provided consent for their data to be included in this study.

## Results

### Baseline characteristics

**Figure 2:**
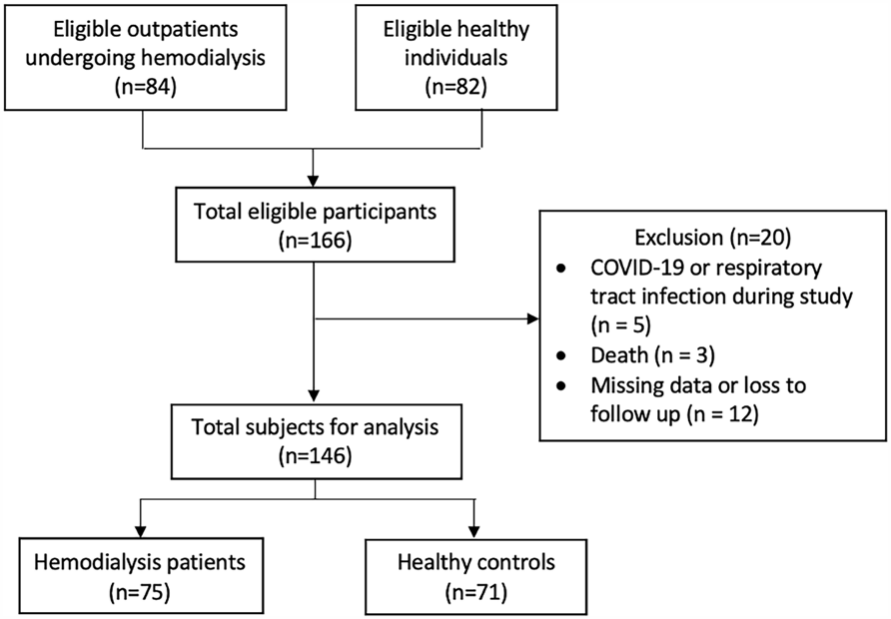
Participant flow chart.

This study included 146 subjects: 75 HD patients and 71 healthy controls (Fig. 2). The baseline characteristics of participants are presented in Table 1. Comorbid diseases of diabetes mellitus, hypertension, and anaemia are significantly higher in proportion among HD patients compared to healthy controls (Diabetes mellitus: 16% vs. 5.6%, p = 0.045; Hypertension: 86.7% vs. 8.3%, p =  < 0.0001; Anaemia: 49.3% vs. 4.2%, p =  < 0.0001). Haemoglobin, lymphocyte count, platelet count, aspartate transaminase (AST), alanine transaminase (ALT), and albumin are significantly lower in HD patients than in healthy controls. Meanwhile, serum blood urea nitrogen (BUN), creatinine, ferritin, and uric acid are significantly higher in HD patients than in healthy controls.

### Immunogenicity

**Figure 3:**
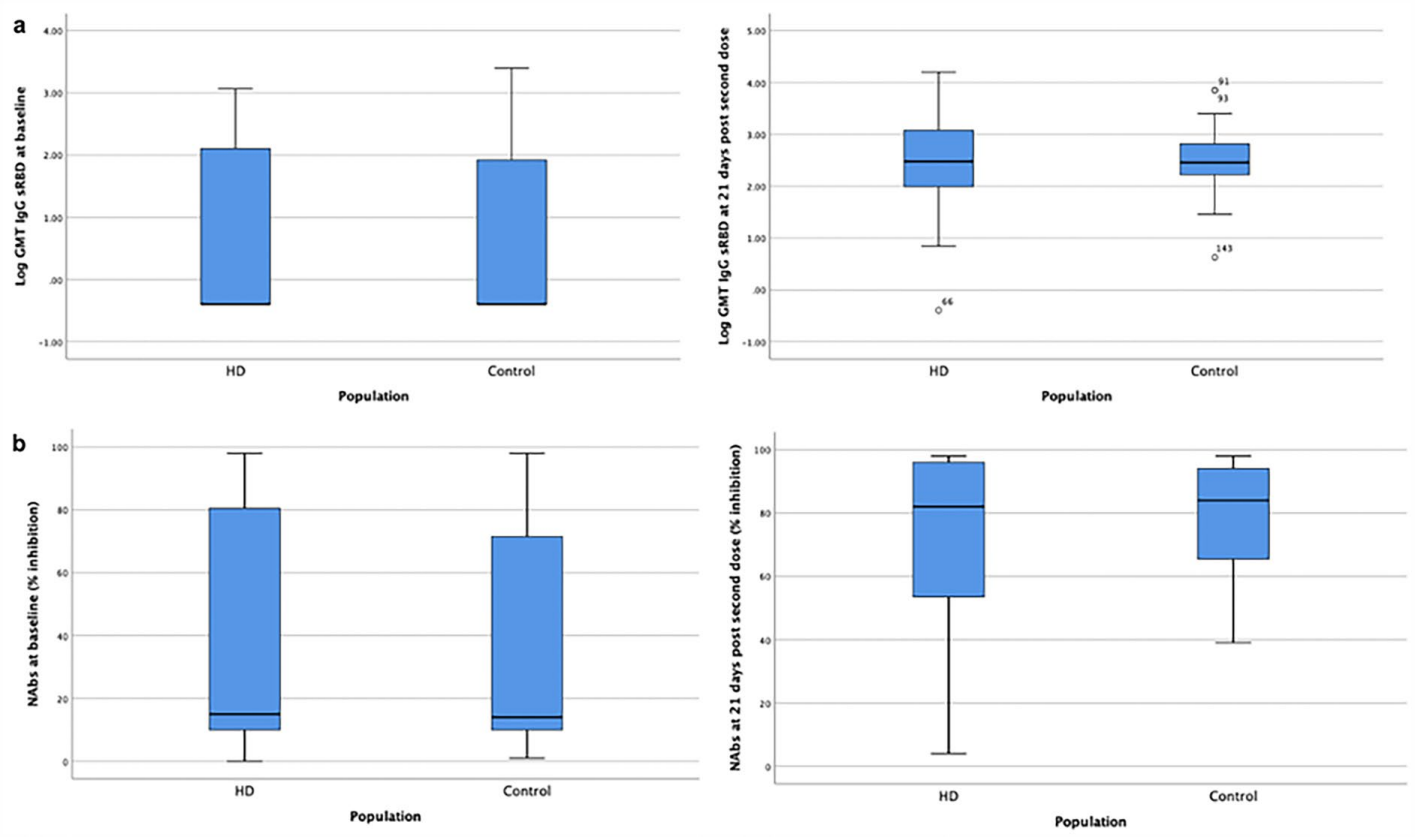
Comparison of (**a**) log Anti-RBD IgG and (**b**) NAbs percentage of inhibition (% inhibition) before vaccination (left) and 21 days after second dose of vaccination (right) between HD patients and healthy controls.

**Figure 4:**
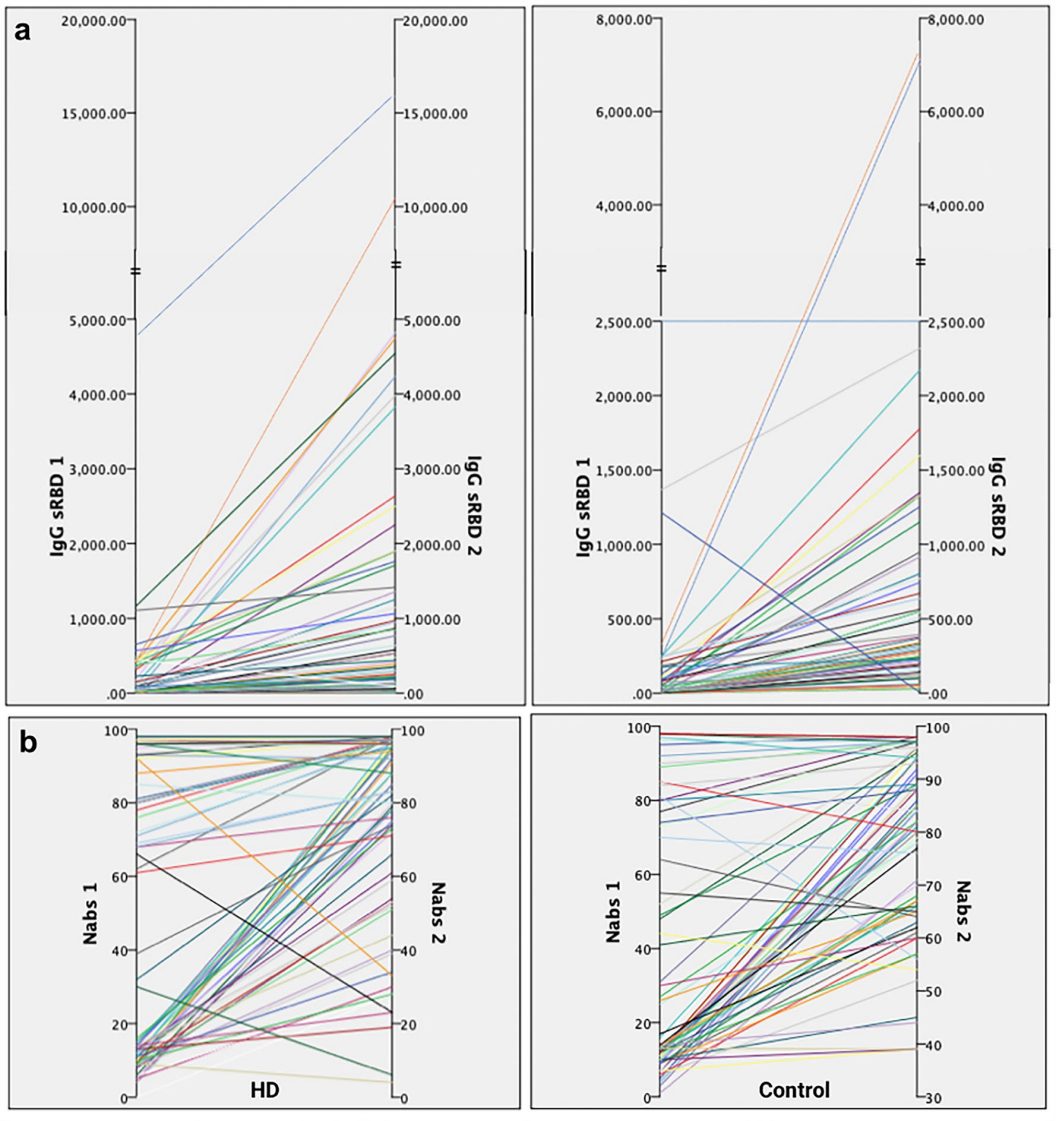
Change in (**a**) anti-RBD IgG titre (U/mL) and (**b**) NAbs percentage of inhibition (%) (left: HD; right: control) at 21 days after two doses of vaccination.

The immunogenicity among participants after inactivated SARS-CoV-2 vaccination is measured with anti-RBD geometric mean titre (GMT), NAbs percentage of inhibition, and seroconversion rates at 21 days after the second dose (Table 2 and Fig. 3). There was no significant difference between both groups in anti-RBD IgG GMT, NAbs percentage of inhibition, and seroconversion rates after the second dose. However, the increase in the inhibition rate of healthy controls (46%) was significantly more significant than in HD patients (25%) (p = 0.049). The change in log anti-RBD IgG and NAbs %inhibition before and after vaccination among both groups is illustrated in Fig. 4.

**Table 2:** Immunogenicity after inactivated SARS-CoV-2 vaccination in HD patients and controls.

Immunogenicity	HD (n = 75)	Control (n = 71)	p value*
Anti-RBD IgG antibody, U/mL			
Baseline IgG RBD, GMT (SD) (U/mL)	4.79 ± 22.08	4.19 ± 18.19	0.789
IgG RBD after second dose, GMT (SD) (U/mL)	277.91 ± 7.13	315.50 ± 3.50	0.645
IgG RBD fold increase, GMT (SD) (U/mL)	57.98 ± 11.32	75.23 ± 12.60	0.527
Seroconversion rate, n (%)	65 (86.7%)	61 (85.9%)	0.895
Neutralizing antibodies, % inhibition			
Baseline NAbs, median (IQR) (%)	15 (10–81)	14 (10–73)	0.359
NAbs after second dose, median (IQR) (%)	82 (53–96)	84 (39–98)	0.654
NAbs increase, median (IQR) (%)	25 (5–63)	46 (10–70)	**0.049**
Seroconversion rate, n (%)	34 (45.3%)	43 (60.6%)	0.065

*GMT* geometric mean titre, *SD* standard deviation.

*Independent t-test or Mann Whitney U test, statistically significant if p value < 0.05 (typed in bold).

Considering that many participants were already seropositive at baseline (Table 1), the immunogenicity measures might be confounded. Therefore, we conducted further analyses to compare HD patients and seronegative controls at baseline (Table 3). The seroconversion rate of NAbs is the only significantly different measure between the HD patients (34/42 or 81%) and controls (43/43 or 100%) (p = 0.003).

**Table 3:** Immunogenicity after inactivated SARS-CoV-2 vaccination in HD patients and controls who were seronegative at baseline.

Immunogenicity	HD	Control	p value*
Anti-RBD IgG antibody, U/mL	n = 65	n = 61	
Baseline IgG RBD, GMT (SD) (U/mL)	2.65 ± 15.84	2.04 ± 11.27	0.571
IgG RBD after second dose, GMT (SD) (U/mL)	265.16 ± 6.39	306.69 ± 3.17	0.600
IgG RBD fold increase, GMT (SD) (U/mL)	99.95 ± 8.24	150.49 ± 6.29	0.249
Seroconversion rate, n (%)	2.65 ± 15.84	2.04 ± 11.27	0.571

Neutralizing antibodies, % inhibition	n = 42	n = 43	
Baseline NAbs, median (IQR) (%)	10 (8–13.25)	11 (9–13)	0.609
NAbs after second dose, median (IQR) (%)	69 (39.75–85)	78 (61–86)	0.056
NAbs increase, median (IQR) (%)	56 (30.75–73)	63 (48–78)	0.091
Seroconversion rate, n (%)	34 (81.0%)	43 (100%)	**0.003**

*GMT* geometric mean titre, *SD* standard deviation.

*Independent t-test or Mann Whitney U test, statistically significant if p value < 0.05 (typed in bold).

### Responders vs. non-responders

Overall, the seroconversion rate among all participants is 86.3% (126/146) and 52.74% (77/146) for NAbs. Vaccination responder rates were respectively 86.7% (65/75) in HD patients and 85.9% (61/71) in healthy controls for anti-RBD IgG antibody, and 45.3% (34/75) in HD patients and 60.6% (43/71) in healthy controls for NAbs. Table 4, compares the clinical and laboratory characteristics between responders and non-responders. Hypertension is significantly associated with a lower seroconversion rate of neutralizing antibodies in the bivariate analysis (p = 0.021). Further multivariate analysis with binary logistic regression is conducted for variables with a p-value of < 0.25, i.e., hypertension, haemoglobin, and platelet count (Table 5). Hypertension, haemoglobin, and platelet count are not significantly different between NAbs responders and non-responders. Meanwhile, this further analysis could not be done for anti-RGB IgD antibody because no variables generated a p-value of < 0.25 in the bivariate analysis.

**Table 4:** Comparison of vaccine responsiveness according to baseline characteristics.

Anti-RBD IgG antibody			
Characteristics	Responder (n = 126)	Non-responder (n = 20)	p value*
Diabetes Mellitus	13 (10.3%)	3 (15%)	0.462
Hypertension	61 (48.4%)	10 (50%)	0.895
Anemia	34 (27%)	6 (30%)	0.779
Hemoglobin, g/dL			
Mean ± SD	11.58 ± 2.59	11.82 ± 3.62	0.717
Lymphocyte count, 10^9^ cells/L			
Mean ± SD	1883.33 ± 695.59	1834.50 ± 723.07	0.772
Platelet count, 10^9^ cells/L			
Mean ± SD	238,007.94 ± 71,989.83	223,700.00 ± 62,382.76	0.403
BUN, mg/dL			
Median (Q1–Q3)	24.65 (10.13–58.05)	21.15 (9.78–69.23)	0.860
Creatinine, mg/dL			
Median (Q1–Q3)	5.71 (0.85–11.02)	3.31 (0.94–15.18)	0.434
AST, U/L			
Median (Q1–Q3)	17 (14–22)	19 (13–24)	0.654
ALT, U/L (n = 133)			
Median (Q1–Q3)	16 (11–24)	17 (12–25)	0.494
Ferritin, ng/mL			
Median (Q1–Q3)	154.16 (55–154.16)	201.06 (121.41–201.06)	0.535
Random Blood Glucose, mg/dL			
Median (Q1–Q3)	96 (88–118)	106 (90–131)	0.265
Albumin, g/dL			
Mean ± SD	4.28 ± 0.48	4.28 ± 0.36	0.997
Uric Acid, mg/dL			
Median (IQR)	5.5 (4.6–7.23)	6.45 (4.73–8.58)	0.266

Neutralizing antibodies (NAbs)			
Characteristics	Responder (n = 77)	Non-responder (n = 69)	p value*
Diabetes Mellitus	8 (10.3%)	8 (11.8%)	0.796
Hypertension	31 (39.7%)	40 (58.8%)	**0.021**
Anemia	19 (24.4%)	21 (30.9%)	0.378
Hemoglobin, g/dL			
Mean ± SD	11.97 ± 2.60	11.20 ± 2.86	0.089
Lymphocyte count, 10^9^ cells/L			
Mean ± SD	1923.12 ± 704.44	1824.78 ± 690.17	0.397
Platelet count, 10^9^ cells/L			
Mean ± SD	245,467.53 ± 71,735.92	225,536.23 ± 68,570.83	0.089
BUN, mg/dL			
Median (Q1–Q3)	15.7 (9.9–55.05)	38.1 (10.25–62.55)	0.860
Creatinine, mg/dL			
Median (Q1–Q3)	1.07 (0.78–0.94)	7.6 (10.92–12.76)	0.434
AST, U/L			
Median (Q1–Q3)	17 (14–20.25)	18 (14–23)	0.582
ALT, U/L (n = 133)			
Median (Q1–Q3)	17 (11–24)	15 (11–24)	0.628
Ferritin, ng/mL			
Median (Q1–Q3)	124.54 (57.5–347.73)	206.07 (72.38–465)	0.535
Random Blood Glucose, mg/dL			
Median (Q1–Q3)	96 (87–115)	100 (90.5–125.5)	0.344
Albumin, g/dL			
Mean ± SD	4.28 ± 0.46	4.28 ± 0.48	0.932
Uric Acid, mg/dL			
Median (IQR)	5.5 (4.6–7.3)	6.1 (4.6–7.3)	0.266

*Pearson’s chi-square or Fisher’s exact for categorical variables, independent t test or Mann Whitney U test for continuous variables, statistically significant if p value < 0.05 (typed in bold).

**Table 5:** Multivariate analysis of significant characteristics for NAbs responders.

Characteristics	p value	Exponent B	95% CI	
			Lower	Upper
Hypertension	0.112	0.484	0.198	1.183
Haemoglobin	0.980	1.002	0.851	1.181
Platelet count	0.364	1.000	1.000	1.000

*Binary logistic regression, statistically significant if p value < 0.05 (typed in bold).

### Adverse events

**Figure 5:**
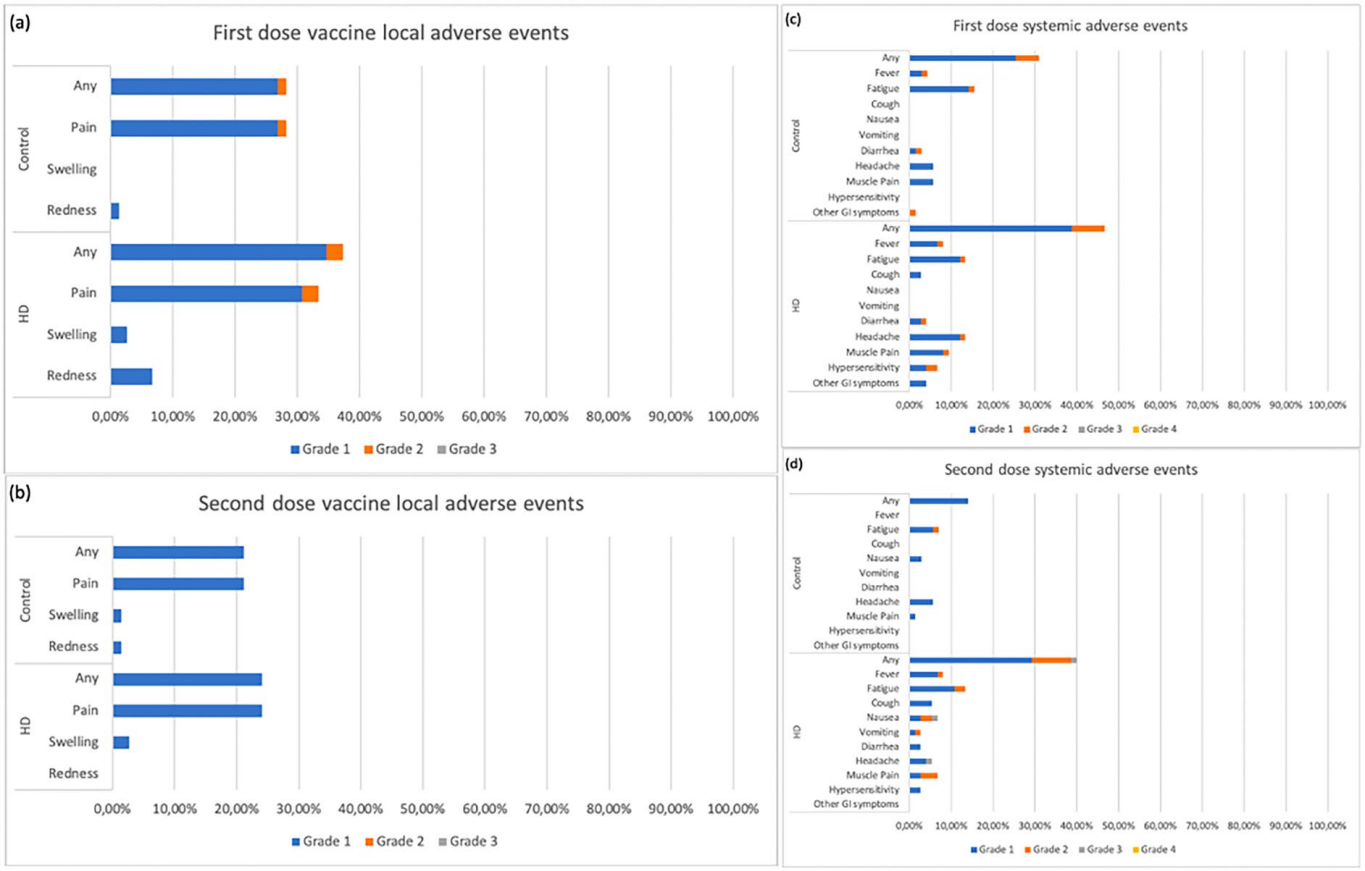
Adverse events after COVID-19 vaccination among HD patients and healthy controls. (**a**) First dose local AEs, (**b**) Second dose local AEs, (**c**) First dose systemic AEs, (**d**) Second dose systemic AEs.

The occurrence of adverse events after the first and second doses of vaccination among HD patients and healthy controls are summarized in Table 6 and Fig. 5. Overall, there is no difference in the number of adverse events between HD patients and healthy controls, except for solicited AEs after the second dose, which was higher among HD patients. The most common AEs included pain at the injection site (21–33%), fatigue (6–16%), headache (5–13%), muscle aches (1–9%), and fever (0–8%) (Fig. 5). The AEs were almost entirely resolved on the 7th day. Almost all AEs were classified as Grade 1 or Grade 2. No serious adverse events were reported among both groups.

**Table 6:** Summary of adverse events.

Adverse events (AEs)	HD (n = 75)	Control (n = 71)	p value*
First dose			
Solicited AEs			
Subjects with at least one solicited AE	43 (57.33%)	34 (47.89%)	0.253
Total no. of solicited AE	78	47	NA
Total no. of severe solicited AE (Grade 3 and 4)	0 (0%)	0 (0%)	NA
Unsolicited AEs			
Subjects with at least one solicited AE	25 (33.33%)	20 (28.17%)	0.499
Total no. of solicited AE	34	28	NA
Total no. of severe solicited AE (Grade 3 and 4)	0 (0%)	0 (0%)	NA
Second dose			
Solicited AEs			
Subjects with at least one solicited AE	39 (52%)	21 (29.58%)	**0.006**
Total no. of solicited AE	57	28	NA
Total no. of severe solicited AE (Grade 3 and 4)	2 (3.51%)	0 (0%)	1.000
Unsolicited AEs			
Subjects with at least one solicited AE	5 (6.67%)	7 (9.86%)	0.483
Total no. of solicited AE	5	7	NA
Total no. of severe solicited AE (Grade 3 and 4)	0 (0%)	0 (0%)	NA

*Pearson’s chi-square or Fisher’s exact, statistically significant if p value < 0.05 (typed in bold).

## Discussion

This study evaluated the immunogenicity and safety after vaccination with inactivated COVID-19 vaccines among haemodialysis patients in comparison with healthy individuals. Antibody levels have been demonstrated to peak around 2–4 weeks after vaccination. We assessed the humoral response three weeks after the second dose since HD patients may have delayed immune response. As expected, in terms of baseline characteristics, several comorbid conditions, including diabetes mellitus, hypertension, and anaemia, are significantly more prevalent in HD patients compared to healthy controls. Diabetes mellitus and hypertension are the leading causes of chronic kidney disease (CKD)^16^. Meanwhile, anaemia is a common complication in CKD, and the prevalence increases with the stage of CKD, from 8.4% at stage 1 to 53.4% at stage 5^17^. All laboratory values are significantly different among both groups owing to the pathophysiological processes and complications involved in compromised kidney function.

Our study demonstrated no significant difference in anti-RBD IgG geometric mean titre, NAbs median inhibition rate, and seroconversion rates after two doses of vaccination with inactivated COVID-19 vaccines (CoronaVac®) between HD patients and healthy individuals. However, several study participants (44% of HD patients and 39.4% of healthy controls) were seropositive before vaccination, which might confound the results. These seropositive subjects might have had previous asymptomatic to mild COVID-19 and were not tested and diagnosed. Being an archipelagic state and the world’s fourth-most populous country, COVID-19 tracing, and testing in Indonesia were not adequate and thorough, especially during the early pandemic. Therefore, we also evaluated the increase in NAbs inhibition rate and found that this value was significantly lower among HD patients than healthy controls. We also conducted further analyses to compare only HD patients and among healthy controls who were seronegative at baseline. In this subgroup, the seroconversion rate of NAbs was significantly lower among HD patients than among healthy controls. These findings demonstrated an adequate but weaker humoral response after vaccination in HD patients than in healthy controls.

Several previous studies have demonstrated similar results. Murt et al. evaluated the immunologic response toward inactivated vaccine (CoronaVac®) and mRNA vaccine (BNT162b2) in haemodialysis patients compared to healthy healthcare workers. The study also demonstrated significantly lower seropositivity rates and antibody levels in haemodialysis patients compared to controls (seropositivity: 80% vs. 99%, p = 0.000; IgG: 408.9 ± 433.5 vs. 685.9 ± 436.9, p = 0.000; NAbs: 58.0 ± 61.5 vs. 97.3 ± 67.0; p = 0.000). Murt et al. involved patients with three times weekly haemodialysis sessions and used the chemiluminescent microparticle immunoassay (CMIA) (Abbott SARS-CoV-2 IgG II Quant, Chicago, USA) to measure the antibody levels^18^. Meanwhile, our study included patients with twice-weekly haemodialysis and utilized the ECLIA. Bai et al. also assessed the immunogenicity after CoronaVac vaccination in 50 HD patients compared to 31 healthy controls. The humoral response was evaluated by measuring the anti-S IgG titer. This study demonstrated a responder rate of 93.5% in healthy controls compared to 94% in the HD group in the third week after the second dose of vaccine (p = 0.93)^19^.

Grupper et al. showed that haemodialysis patients developed a substantial but lower humoral response following the BNT162b2/Pfizer vaccine. All subjects in the control group (100%) comprising healthcare workers developed an antibody response compared with 96% in the dialysis group. In this study, the IgG levels in the dialysis group (2900 [1128-5651] IU/mL) were significantly lower than in the control group (7401 [3687-15471] IU/mL) (p < 0.001)^20^. Another study by Fu et al. examined the humoral responses of 385 HD patients compared to healthcare workers receiving complete two-dose ChAdOx1 nCoV-19 vaccines. Anti-SARS-CoV2 antibodies were measured 4 weeks after the second dose. The antibody levels of HD patients were similar to those of healthcare workers (602.00 [307.50-1623.00] U/mL vs 602.50 [391.25-1029.25], p = 0.814). The seroconversion rate among HD patients was 98.96%, whereas that of healthy controls was 100%^21^.

Our study also demonstrated that no specific clinical or laboratory characteristics are associated with vaccine responsiveness. This is inconsistent with several previous studies’ findings, which reported lower antibody response and vaccine effectiveness in individuals with hypertension^19,22–24^. Recent evidence suggests that certain types of hypertension may be associated with the adaptive immune system. For instance, a significant change in T cell immunometabolism can modulate the metabolic processes and eventually lead to aberrant T cell activation, differentiation, and proliferation, which contribute to the pathogenesis of hypertension^25–27^. In previous studies, the immunogenicity of COVID-19 vaccines has been demonstrated to be lower among patients with diabetes mellitus^19,28^.

Overall, the occurrence of adverse events after vaccination is not significantly different between HD patients and healthy controls, except for the second dose solicited AEs rate being higher among HD patients. This finding contradicts several previous studies, demonstrating that adverse events rates are higher in healthy individuals^29^. Simon et al. revealed a significantly higher number of local and systemic adverse events in healthy controls than in HD patients after both vaccine doses. Similar to our finding, no serious adverse events were reported in either group^30^. Park et al. demonstrated that the proportion of participants with injection site swelling, heat sensation at the injection site, headache, and fatigue were higher within seven days of the first dose among healthy people than among HD patients. Meanwhile, there was no significant difference in adverse events after the second dose between HD patients and healthy people^31^. Similarly, Kolb et al. also reported no substantial differences in adverse events between HD patients and healthy controls after vaccination with BNT162b2 or mRNA-1273^32^.

The strengths of our study are the prospective cohort design and the use of inactivated COVID-19 vaccine when many other studies utilize mRNA or vector-based vaccines. This study is possibly one of the few studies examining the immune response in haemodialysis patients after inactivated SARS-CoV-2 vaccination. Our study had several limitations, including a relatively small number of participants and the inability to appropriately exclude all participants with past SARS-CoV-2 infections due to inadequate tracing and testing in Indonesia during the early pandemic. Past SARS-CoV-2 infections might confound the level of immunity mounted by the vaccine recipients. The study was conducted at only four centres in the Special Region of Yogyakarta and Central Java. Therefore, the results of this study cannot be generalized to all HD populations. Moreover, the sVNT is a surrogate test for neutralizing antibodies, which is less standardized than the plaque reduction neutralization test. Hence, we cannot unequivocally conclude the assumption of protective immunity against infection after this vaccine regimen.

The results of our study suggest that further studies on the effect of a booster dose should be conducted to promote a more substantial and persistent antibody response in HD patients. In addition, patients with low or no response might benefit from regular antibody measurement and more intensive vaccination schedules.

## Conclusions

After two doses of inactivated SARS-CoV-2 vaccination, we report an adequate antibody response among haemodialysis patients. Inactivated SARS-CoV-2 vaccine can be safely administered to haemodialysis patients with tolerable adverse events.

## Data Availability

The datasets generated and analysed during the current study are not publicly available because of privacy and ethical restrictions. Still, anonymized data are available from the corresponding author at reasonable request.
